# Exploring the Role of Oxidative Stress in Skeletal Muscle Atrophy: Mechanisms and Implications

**DOI:** 10.7759/cureus.42178

**Published:** 2023-07-20

**Authors:** Suyash Agrawal, Swarupa Chakole, Nidhi Shetty, Roshan Prasad, Tejaswee Lohakare, Mayur Wanjari

**Affiliations:** 1 Medicine, Jawaharlal Nehru Medical College, Datta Meghe Institute of Higher Education and Research, Wardha, IND; 2 Community Medicine, Jawaharlal Nehru Medical College, Datta Meghe Institute of Higher Education and Research, Wardha, IND; 3 Internal Medicine, Jawaharlal Nehru Medical College, Datta Meghe Institute of Higher Education and Research, Wardha, IND; 4 Child Health Nursing, Smt. Radhikabai Meghe Memorial College of Nursing, Wardha, IND; 5 Research and Development, Jawaharlal Nehru Medical College, Datta Meghe Institute of Higher Education and Research, Wardha, IND

**Keywords:** therapeutic interventions, antioxidant supplementation, muscle regeneration, redox signaling, protein degradation, reactive oxygen species (ros), oxidative stress, skeletal muscle atrophy

## Abstract

Skeletal muscle atrophy is a complex physiological process characterized by progressive muscle mass and strength loss. It is associated with various health conditions, including aging, disease, and certain diseases. Emerging research has indicated that oxidative stress plays a significant role in developing and progressing skeletal muscle atrophy. This review article explores the mechanisms by which oxidative stress influences skeletal muscle atrophy and its implications for potential therapeutic interventions. The review begins by providing an overview of skeletal muscle atrophy and the current understanding of its underlying mechanisms, highlighting the intricate balance between protein degradation and synthesis pathways. Subsequently, the concept of oxidative stress is introduced, discussing its sources and the intricate redox signaling pathways present in skeletal muscle cells. This review's main focus is exploring the multifaceted role of oxidative stress in skeletal muscle atrophy. The detrimental effects of excessive reactive oxygen species (ROS) production on cellular components, including proteins, lipids, and deoxyribonucleic acid (DNA), are discussed. In addition, the impact of oxidative stress on key signaling pathways involved in muscle wasting, such as the ubiquitin-proteasome system and autophagy, is examined. Furthermore, the review highlights the implications of oxidative stress in modulating muscle regeneration and the importance of redox balance in maintaining muscle health. Potential therapeutic strategies targeting oxidative stress, such as antioxidant supplementation, exercise interventions, and pharmacological approaches, are also discussed. In conclusion, this review comprehensively explains the intricate relationship between oxidative stress and skeletal muscle atrophy. By elucidating the underlying mechanisms and discussing potential therapeutic interventions, this review aims to contribute to the development of novel strategies for mitigating muscle wasting and improving overall muscle health.

## Introduction and background

Skeletal muscle atrophy is a complex physiological process characterized by losing muscle mass and strength, often leading to functional impairment and decreased quality of life. It can arise from various conditions, such as aging and chronic diseases. Understanding the underlying mechanisms of skeletal muscle atrophy is essential for developing effective therapeutic strategies to mitigate its impact [1,2]. Skeletal muscle atrophy is the progressive loss of muscle mass commonly associated with a decline in muscle strength. It can occur due to multiple factors, including aging; chronic diseases, such as cancer, heart failure, and chronic obstructive pulmonary disease (COPD); and prolonged periods of inactivity or immobilization. Skeletal muscle atrophy impairs physical performance and contributes to metabolic dysfunction, impaired glucose homeostasis, and reduced overall health [3,4].

Oxidative stress is a state of imbalance between the production of reactive oxygen species (ROS) and the ability of the body's antioxidant defense systems to neutralize them. ROS are highly reactive molecules that can damage cellular components, including proteins, lipids, and deoxyribonucleic acid (DNA). Under normal physiological conditions, a certain level of ROS is produced by cellular metabolism. However, when the production of ROS exceeds the body's antioxidant capacity, oxidative stress occurs [5]. Oxidative stress has been implicated in various pathological conditions, including neurodegenerative diseases, cardiovascular diseases, and cancer. Recent evidence suggests that oxidative stress also plays a critical role in skeletal muscle health and disease. Excessive ROS production and impaired antioxidant defense mechanisms have been observed in skeletal muscle under atrophy conditions [6].

The purpose of this review article is to provide a comprehensive overview of the role of oxidative stress in skeletal muscle atrophy. We aim to explore the mechanisms through which oxidative stress contributes to muscle wasting and discuss the implications of oxidative stress in various contexts, including age-related muscle loss (sarcopenia), chronic diseases associated with muscle wasting (e.g., cancer cachexia), and disuse-induced muscle atrophy (e.g., immobilization and bed rest). We aim to elucidate the intricate interplay between oxidative stress and skeletal muscle atrophy by synthesizing the existing literature. In addition, we will discuss the potential therapeutic strategies that target oxidative stress to mitigate muscle wasting and improve muscle health.

## Review

Oxidative stress in the skeletal muscle

Definition and Causes of Oxidative Stress

Oxidative stress is an imbalance between producing ROS and the body's ability to detoxify and repair the damage. ROS encompass a group of highly reactive molecules, including superoxide anion (O_2_·-), hydrogen peroxide (H_2_O_2_), and hydroxyl radical (·OH). Reactive nitrogen species (RNS), such as nitric oxide (NO) and peroxynitrite (ONOO-), also contribute to oxidative stress [7].

Several factors can lead to oxidative stress in the skeletal muscle. These include excessive ROS/RNS production, reduced antioxidant defense systems, mitochondrial dysfunction, inflammation, and disturbances in redox signaling pathways. In addition, various conditions, such as aging, chronic diseases, physical inactivity, and exposure to environmental toxins, can contribute to increased oxidative stress in the skeletal muscle [6].

Overview of ROS and RNS

ROS and RNS are highly reactive molecules derived from oxygen and nitrogen, respectively. While they serve critical physiological functions as signaling molecules in cellular processes, excessive levels or dysregulation of ROS and RNS can cause oxidative damage. ROS include O_2_·-, H_2_O_2_, and ·OH, while RNS include NO and ONOO- [8].

ROS and RNS can participate in redox reactions with cellular components, including proteins, lipids, and DNA, leading to oxidative damage and subsequent dysfunction. Significantly, these reactive species can modulate cellular signaling pathways and gene expression, exerting beneficial and detrimental effects on the skeletal muscle [8].

Sources of ROS and RNS in the Skeletal Muscle

In the skeletal muscle, generating ROS and RNS involves multiple sources. These sources contribute to the overall oxidative stress observed in the skeletal muscle, particularly during conditions of muscle atrophy. The primary sources of ROS and RNS in the skeletal muscle include mitochondria, nicotinamide adenine dinucleotide phosphate (NADPH) oxidases (NOX), xanthine oxidase, and uncoupled nitric oxide synthase (NOS) isoforms [9].

Mitochondria: Mitochondria are the primary site of cellular energy production through oxidative phosphorylation. However, a small fraction of electrons leak from the electron transport chain during this process, generating superoxide (O2•-) as a byproduct. Mitochondrial ROS production can increase significantly under conditions of mitochondrial dysfunction, such as impaired electron transport chain activity or decreased antioxidant defense systems [10].

NOX: NOX are a family of enzymes responsible for generating ROS by transferring electrons from NADPH to molecular oxygen. In the skeletal muscle, NOX enzymes, particularly NOX2 and NOX4 isoforms, are expressed and can contribute to ROS production. The activation of NOX enzymes in response to various stimuli, such as inflammatory cytokines or mechanical stress, can increase ROS levels in skeletal muscle [11].

Xanthine oxidase: Xanthine oxidase is an enzyme involved in purine metabolism. It can generate superoxide when it catalyzes the conversion of hypoxanthine to xanthine and further to uric acid. Increased xanthine oxidase activity has been observed in various pathological conditions and can contribute to ROS production in the skeletal muscle [12].

Uncoupled NOS isoforms: NOSs are enzymes responsible for synthesizing NO, an important signaling molecule. However, under certain conditions, NOS enzymes can become "uncoupled," producing superoxide instead of NO. Uncoupled NOS isoforms, particularly inducible NOS (iNOS), can increase ROS and RNS production in the skeletal muscle [13].

Oxidative Damage and its Impact on Cellular Components

Oxidative stress can result in oxidative damage to various cellular components within the skeletal muscle. Proteins are susceptible to oxidation, leading to their structure and function modifications. These modifications include oxidation of amino acid residues, protein cross-linking, and carbonylation. Oxidative damage to proteins can impair their enzymatic activity, disrupt cellular signaling pathways, and contribute to protein degradation [14].

Lipids, particularly unsaturated fatty acids, are also vulnerable to oxidative damage. ROS can initiate lipid peroxidation, producing reactive lipid species, such as malondialdehyde (MDA) and 4-hydroxynonenal (4-HNE). These reactive lipid species can further damage cellular membranes, affecting their integrity and fluidity [15]. Moreover, DNA damage can occur due to oxidative stress. ROS can directly interact with DNA, leading to the formation of DNA adducts and strand breaks. Accumulated DNA damage can impair gene expression and genomic stability, potentially contributing to cellular dysfunction and apoptosis.

Skeletal muscle atrophy: overview and mechanisms

Definition and Types of Skeletal Muscle Atrophy

Skeletal muscle atrophy is the loss of muscle mass and fiber size, decreasing muscle strength and function. It can be classified into two main types: disuse atrophy and pathological atrophy. Disuse atrophy occurs due to prolonged periods of inactivity or immobilization, such as bed rest or limb immobilization. Meanwhile, pathological atrophy is associated with various diseases and conditions, including aging (sarcopenia), chronic diseases (cancer cachexia and heart failure), and systemic inflammatory response (sepsis) [16].

Overview of the Molecular Pathways Involved in Muscle Atrophy

Muscle atrophy is a complex process involving multiple molecular pathways. The two primary mechanisms implicated in muscle wasting are increased protein breakdown (catabolism) and decreased protein synthesis (anabolism). Several signaling pathways and regulatory factors are involved in these processes [17].

One of the key pathways involved in muscle atrophy is the ubiquitin-proteasome system (UPS). The UPS tags proteins for degradation by attaching ubiquitin molecules to them. The activation of specific E3 ubiquitin ligases, such as muscle-specific RING finger proteins (MuRF1 and MAFbx/atrogin-1), targets muscle proteins for degradation via the proteasome [18]. Another important pathway is the autophagy-lysosome system, which involves the sequestration and degradation of cellular components, including proteins and organelles. Autophagy is upregulated in muscle atrophy and can contribute to protein breakdown.

Key Signaling Pathways and Factors Implicated in Muscle Wasting

Multiple signaling pathways and factors contribute to muscle wasting. The nuclear factor-kappa B (NF-κB) pathway, a key regulator of inflammation, is activated in muscle atrophy. NF-κB signaling induces the expression of pro-inflammatory cytokines, such as tumor necrosis factor-alpha (TNF-α) and interleukin-6 (IL-6), which can promote muscle protein degradation [19].

The myostatin signaling pathway, a negative regulator of muscle growth, is also implicated in muscle atrophy. Increased myostatin levels suppress muscle protein synthesis and promote protein breakdown, leading to muscle wasting [20].

Insulin-like growth factor 1 (IGF-1) and the mammalian target of the rapamycin (mTOR) pathway are crucial for muscle growth and maintenance. Reduced IGF-1 signaling and mTOR activity contribute to muscle atrophy by impairing protein synthesis and promoting protein degradation [21]. Other factors, such as glucocorticoids (e.g., cortisol), pro-inflammatory cytokines, oxidative stress, and mitochondrial dysfunction, play significant roles in muscle wasting by influencing various signaling pathways and processes.

Role of Inflammation in Skeletal Muscle Atrophy

Inflammation plays a prominent role in developing and progressing skeletal muscle atrophy. In various conditions associated with muscle wasting, including aging, chronic diseases, and disuse, inflammation contributes to the deterioration of muscle tissues. The interplay between inflammatory mediators, immune cells, and muscle fibers leads to a cascade of events that promote muscle catabolism and impair muscle regeneration [22].

One crucial aspect of inflammation in muscle atrophy is the rise in pro-inflammatory cytokines, which includes TNF-α and IL-6. These cytokines are known to increase in conditions associated with muscle wasting and can be produced by infiltrating immune cells, such as macrophages and muscle fibers. Elevated levels of TNF-α and IL-6 contribute to muscle protein breakdown and hinder protein synthesis, resulting in muscle loss [23].

The activation of signaling pathways plays a significant role in the mechanism by which pro-inflammatory cytokines induce muscle atrophy. In particular, the NF-κB signaling pathway is closely involved in upregulating various genes associated with muscle proteolysis, including the UPS and autophagy. The activation of NF-κB facilitates the degradation of muscle proteins, ultimately leading to muscle wasting [24].

Furthermore, the relationship between TNF-α, IL-1, and NF-κB deserves attention regarding their collective impact on muscle wasting. TNF-α and IL-1 can activate NF-κB signaling, amplifying the downstream effects on muscle proteolysis. The combined action of TNF-α, IL-1, and NF-κB enhances the expression of genes associated with muscle protein breakdown, thereby exacerbating muscle wasting [25]. By elucidating the interplay between TNF-α, IL-1, and NF-κB, it becomes evident that their concerted effect on muscle wasting involves regulating muscle proteolysis pathways and inhibiting protein synthesis. Understanding these relationships provides valuable insights into the complex mechanisms underlying muscle atrophy and highlights potential therapeutic targets for mitigating muscle loss associated with inflammatory conditions.

In addition to cytokines, infiltrating immune cells, particularly macrophages, play a significant role in inflammation-driven muscle atrophy. During muscle wasting, macrophages are recruited to the site of injury or damage. These immune cells release factors contributing to muscle catabolism, including cytokines, chemokines, and ROS. Cytokines, such as TNF-α and IL-6, produced by macrophages further propagate the inflammatory response, amplifying muscle protein degradation [25].

Moreover, chronic inflammation in skeletal muscle can impair the regenerative capacity of muscle tissues. Inflammatory processes disrupt the balance between muscle fiber breakdown and regeneration, leading to an imbalance favoring muscle loss. Prolonged inflammation can also promote fibrosis, characterized by excessive collagen and connective tissue deposition, further exacerbating muscle wasting and impairing muscle function [26]. Understanding the role of inflammation in skeletal muscle atrophy provides insights into the underlying mechanisms of muscle wasting. Targeting inflammatory pathways and mitigating chronic inflammation may offer potential therapeutic strategies for preventing or reversing muscle atrophy. Modulating the production or activity of pro-inflammatory cytokines, inhibiting NF-κB signaling, and promoting an anti-inflammatory environment are among the approaches that hold promise in attenuating muscle wasting and preserving muscle health.

Linking oxidative stress and skeletal muscle atrophy

Oxidative Stress as a Contributing Factor to Muscle Wasting

Oxidative stress has emerged as a contributing factor in skeletal muscle atrophy. Excessive production of ROS and RNS can promote muscle wasting through various mechanisms. Elevated oxidative stress levels can disrupt cellular homeostasis, leading to protein degradation, impaired protein synthesis, altered cellular signaling, and mitochondrial dysfunction, all contributing to muscle atrophy [27].

Impact of ROS and RNS on Muscle Protein Synthesis and Breakdown

ROS and RNS can directly influence muscle protein synthesis and breakdown. Oxidative stress can inhibit protein synthesis by affecting translation initiation factors, reducing ribosomal activity, and impairing the function of key signaling molecules involved in protein synthesis, such as mTOR. In addition, ROS/RNS can activate signaling pathways, such as the NF-κB pathway, which promote the expression of specific E3 ubiquitin ligases (e.g., MuRF1 and MAFbx/atrogin-1) responsible for protein degradation via the UPS [28].

Oxidative Stress-Induced Disruption of Cellular Signaling Pathways

Oxidative stress can disrupt cellular signaling pathways involved in muscle homeostasis. ROS/RNS can modify and oxidize critical components of signaling cascades, leading to aberrant signaling. For instance, oxidative stress can activate the NF-κB pathway, producing pro-inflammatory cytokines that promote muscle protein breakdown. Furthermore, oxidative stress can modulate the IGF-1/mTOR pathway, impair insulin signaling, and perturb other growth-related signaling pathways, all contributing to muscle wasting [29].

Mitochondrial Dysfunction and Its Relationship to Oxidative Stress and Muscle Atrophy

Mitochondria play a crucial role in cellular energy production and are a major source of ROS generation. Oxidative stress can disrupt mitochondrial function, leading to mitochondrial dysfunction. Dysfunctional mitochondria produce more ROS, exacerbating oxidative stress and creating a vicious cycle. Mitochondrial dysfunction can impair ATP production, induce oxidative damage to mitochondrial DNA, compromise calcium homeostasis, and impair cellular respiration, contributing to muscle atrophy [30]. Moreover, mitochondrial dysfunction and oxidative stress can impair autophagy, as redox-sensitive signaling pathways regulate autophagy-related genes. Dysregulated autophagy can disrupt cellular protein and organelle turnover, further contributing to muscle wasting. The relationship between oxidative stress, mitochondrial dysfunction, and muscle atrophy is bidirectional. Oxidative stress can induce mitochondrial dysfunction, while dysfunctional mitochondria generate more ROS, perpetuating oxidative stress and muscle wasting.

Implications of oxidative stress in skeletal muscle atrophy

Role of Oxidative Stress in Age-Related Muscle Loss (Sarcopenia)

Oxidative stress has been implicated in developing and progressing age-related muscle loss, known as sarcopenia. During aging, oxidative damage in skeletal muscle accumulates due to an imbalance between ROS production and antioxidant defense systems. Increased oxidative stress can contribute to mitochondrial dysfunction, impaired protein synthesis, and enhanced protein breakdown, all of which play a role in sarcopenia. Targeting oxidative stress may offer potential therapeutic interventions to attenuate age-related muscle loss [31].

Oxidative Stress and Muscle Wasting in Chronic Diseases

Chronic diseases, such as cancer, are often accompanied by muscle wasting, a condition known as cachexia. Oxidative stress plays a significant role in the pathogenesis of cancer cachexia. Tumor-derived factors, inflammatory cytokines, and increased metabolic demands associated with cancer promote oxidative stress in skeletal muscle. Oxidative stress can trigger muscle protein degradation, impair protein synthesis, and contribute to muscle fiber atrophy. Modulating oxidative stress levels may hold promise in ameliorating muscle wasting in chronic diseases, such as cancer cachexia [32].

Oxidative Stress in Disuse-Induced Muscle Atrophy

Disuse-induced muscle atrophy occurs during prolonged immobilization or bed rest, such as during hospitalization or spaceflight. Oxidative stress is implicated in the development of disuse-induced muscle atrophy. Reduced physical activity leads to increased ROS production and decreased antioxidant capacity in the skeletal muscle. Oxidative stress can promote protein degradation, impair protein synthesis, and disrupt cellular signaling pathways involved in muscle maintenance. Managing oxidative stress may provide a therapeutic avenue to mitigate disuse-induced muscle atrophy [33].

Potential Therapeutic Strategies Targeting Oxidative Stress to Mitigate Muscle Wasting

Given the significant role of oxidative stress in skeletal muscle atrophy, targeting oxidative stress through various strategies holds promise as a therapeutic approach to mitigate muscle wasting and promote muscle health. These strategies include:

Antioxidant supplementation: Administration of exogenous antioxidants can help counteract oxidative stress and reduce muscle wasting. Antioxidants, such as vitamins C and E or natural antioxidants found in fruits and vegetables, can scavenge free radicals and prevent oxidative damage to muscle tissues [34].

Exercise and physical activity: Exercise and physical activity are crucial in slowing age-related muscular atrophy (sarcopenia). It is important to explicitly mention that regular exercise and physical activity have been shown to enhance the antioxidant defense systems in skeletal muscle, thereby combating the effects of sarcopenia. Exercise provides significant benefits by stimulating the production of endogenous antioxidants, improving mitochondrial function, and reducing oxidative stress. In particular, structured exercise programs tailored to specific populations experiencing muscle atrophy, such as resistance training or aerobic exercise, have been proven effective in mitigating oxidative stress and preserving muscle mass [35].

Pharmacological interventions: Pharmacological agents that target oxidative stress pathways offer potential therapeutic options. These interventions can include compounds that enhance the activity of endogenous antioxidant enzymes, such as superoxide dismutase (SOD) or glutathione peroxidase (GPx), or molecules that modulate redox signaling pathways. By enhancing the antioxidant defense mechanisms or suppressing ROS generation, these agents may attenuate oxidative stress and its detrimental effects on muscle health [36]. Although the abbreviations SOD, GPx, and ROS are expanded further down in the experimental approach section, it is recommended to include their full expansions before using the abbreviations for the first time [36].

Nutritional interventions: Dietary strategies that provide an optimal balance of macronutrients and include antioxidant-rich foods can help reduce oxidative stress in the skeletal muscle. Consuming a diet rich in fruits, vegetables, whole grains, and lean proteins provides a variety of antioxidants that can scavenge free radicals and protect against oxidative damage. Modulating specific nutrient-sensing pathways, such as the mTOR or sirtuins, through dietary interventions may also influence oxidative stress and promote muscle health [37].

Lifestyle modifications: Healthy lifestyles can minimize oxidative stress and promote overall muscle health. Adequate sleep, stress reduction techniques, and avoidance of environmental toxins can help reduce the burden of oxidative stress on the skeletal muscle. Ensuring sufficient rest and recovery, managing stress levels, and minimizing exposure to pollutants and toxins can contribute to maintaining a balanced redox state in muscle tissues [38]. It is important to note that the effectiveness of these strategies may vary depending on the underlying cause and context of muscle atrophy. Further research is needed to elucidate the optimal approaches and their specific applications in different populations and disease conditions. In addition, personalized and multi-modal approaches that combine multiple strategies may synergistically reduce oxidative stress and preserve muscle mass.

Experimental approaches and assessment of oxidative stress in the skeletal muscle

Methods for Measuring Oxidative Stress Markers in Muscle Tissues

Measurement of ROS and RNS: Techniques, such as dihydroethidium (DHE) staining, dichlorofluorescein (DCF) assay, or electron paramagnetic resonance (EPR) spectroscopy, can be used to detect and quantify ROS and RNS levels in muscle tissues. DHE staining involves using a fluorescent probe that becomes oxidized upon exposure to ROS, producing a fluorescent signal that can be visualized and quantified. DCF assay utilizes a non-fluorescent probe that is converted into a fluorescent compound in the presence of ROS, allowing for the measurement of ROS levels. EPR spectroscopy measures the unpaired electrons of free radicals, providing quantitative information about ROS and RNS levels [39].

Assessment of lipid peroxidation: Lipid peroxidation, which is a result of oxidative damage to lipids, can be assessed by measuring MDA levels or by using specific probes, such as the thiobarbituric acid reactive substance (TBARS) assay. MDA is a byproduct of lipid peroxidation and can be measured using spectrophotometric or chromatographic methods. The TBARS assay detects the presence of MDA by reacting with thiobarbituric acid, forming a colored complex that can be quantified spectrophotometrically. These methods indicate the extent of lipid peroxidation in muscle tissues, reflecting oxidative damage [40].

Evaluation of antioxidant enzyme activity: The activity of antioxidant enzymes, such as superoxide dismutase (SOD), catalase, and GPx, can be measured to assess the antioxidant defense capacity in muscle tissues. SOD catalyzes the dismutation of superoxide radicals, catalase converts hydrogen peroxide into water and oxygen, and GPx reduces hydroperoxides using glutathione (GSH) as a cofactor. The activity of these enzymes can be determined through various spectrophotometric or fluorometric assays, providing insights into the ability of the muscle tissues to neutralize ROS and maintain redox balance [41].

Measurement of antioxidant molecule levels: Levels of non-enzymatic antioxidants, such as GSH and vitamin C, can be determined to evaluate the antioxidant status in the skeletal muscle. GSH is a tripeptide that is a major cellular antioxidant, and vitamin C (ascorbic acid) is a water-soluble antioxidant. GSH and vitamin C levels can be measured using various biochemical assays, such as spectrophotometric or chromatographic methods. These measurements reflect the antioxidant capacity and provide information about the ability of muscle tissues to scavenge and neutralize free radicals [42].

Animal models and in vitro systems used to study oxidative stress and muscle atrophy

Animal Models

Rodents, such as mice and rats, are commonly used animal models in studying skeletal muscle atrophy and oxidative stress. These models offer several advantages for investigating the complex relationship between oxidative stress and muscle wasting. Rodents share many physiological and genetic similarities with humans, making them suitable for translational research. They also have well-characterized muscle anatomy and physiology, facilitating studying muscle-specific changes. Rodent models allow for manipulating various factors contributing to muscle atrophy, including disuse, aging, immobilization, denervation, inflammation, and chronic diseases, such as cancer. By manipulating these factors, researchers can assess the impact of oxidative stress on muscle health and explore potential therapeutic interventions [43].

Animal models provide opportunities for in vivo measurements of oxidative stress markers and functional assessments of muscle strength, contractile properties, and morphology. Techniques, such as histological analysis, immunohistochemistry, biochemical assays, and molecular analysis, can examine oxidative stress markers, protein expression, gene expression, and signaling pathways involved in muscle atrophy. In addition, studies in animal models allow for the evaluation of systemic factors, such as circulating cytokines and hormones, which can contribute to oxidative stress and muscle wasting [44].

Cell Culture Systems

In vitro systems using muscle cell lines or primary muscle cells offer controlled experimental conditions to investigate the effects of oxidative stress on muscle atrophy. These systems provide a simplified yet controlled environment that enables researchers to focus on specific cellular and molecular mechanisms involved in oxidative stress-induced muscle wasting [45].

Muscle cell lines, such as C2C12 myoblasts, are commonly used to study muscle biology and the effects of oxidative stress. These immortalized cell lines offer the advantage of continuous availability and ease of maintenance. Primary muscle cells isolated from animal models or human biopsies provide a more physiologically relevant model. These primary cells can be differentiated into myotubes, resembling mature muscle fibers and allowing researchers to investigate the effects of oxidative stress on muscle structure and function [46].

In cell culture systems, oxidative stress can be induced through various methods, such as exposure to ROS-generating compounds or manipulating antioxidant defense systems. Using fluorescent probes, enzymatic assays, and immunostaining, researchers can assess oxidative stress markers, intracellular ROS levels, antioxidant enzyme activities, and cellular redox status. Molecular techniques, including gene expression analysis, protein quantification, and signaling pathway studies, can provide insights into the mechanisms by which oxidative stress contributes to muscle atrophy [47].

Cell culture systems offer the advantage of experimental control and the ability to perform high-throughput studies. However, it is essential to recognize the limitations of these systems, such as the absence of the complex in vivo microenvironment and the potential differences between cultured cells and intact muscle tissues. Therefore, findings from cell culture studies should be interpreted in conjunction with in vivo experiments to ensure their physiological relevance [48].

Limitations and Challenges in Studying Oxidative Stress in the Skeletal Muscle

Quantification and interpretation of oxidative stress markers: Assessing oxidative stress in the skeletal muscle is challenging due to the complex nature of oxidative stress and basal levels of ROS. Multiple markers are utilized to evaluate oxidative stress, including ROS/RNS levels, lipid peroxidation products, and antioxidant enzyme activities. However, accurately quantifying and interpreting these markers require careful analysis. The dynamic nature of oxidative stress adds further complexity, as the levels can vary depending on the physiological state of the muscle. Longitudinal measurements and considering temporal changes in oxidative stress markers are necessary to capture the full picture and understand their role in muscle atrophy [49].

Specificity of oxidative stress markers: Not all oxidative stress markers are specific to muscle tissues, and their measurement can be influenced by factors unrelated to muscle atrophy. For example, systemic factors, such as inflammation or other comorbidities, can impact oxidative stress markers in circulation. Therefore, careful consideration should be given to the specificity and relevance of chosen markers when assessing oxidative stress in the skeletal muscle. It is crucial to select markers validated for muscle-specific oxidative stress and to interpret the results in the context of muscle-specific mechanisms [50].

Standardization of experimental protocols: Experimental protocol variations can introduce variability and make comparisons between studies challenging. Sample collection, tissue processing, and measurement techniques can significantly influence the results. Therefore, it is crucial to standardize experimental protocols to ensure consistency and reproducibility of results. Standardization efforts should encompass guidelines for sample handling, storage conditions, and validated measurement techniques. Collaborative initiatives and establishing consensus guidelines can facilitate the standardization process in oxidative stress research in the skeletal muscle [51].

Translatability to human conditions: Animal models and in vitro systems provide valuable insights into oxidative stress and muscle atrophy. However, translating findings to human skeletal muscle atrophy requires careful consideration due to potential species differences and limitations in modeling complex human diseases accurately. The physiological and molecular characteristics of skeletal muscle may differ between species, and the underlying mechanisms of muscle wasting can vary. Therefore, findings from animal and in vitro studies should be cautiously extrapolated to human conditions. Complementary research involving human subjects and relevant disease models is necessary to bridge the gap between preclinical and clinical observations [52].

Dynamic nature of oxidative stress: Oxidative stress is a dynamic process influenced by various factors, including metabolic demands, exercise, inflammation, and disease conditions. Measuring oxidative stress simultaneously may not fully capture the temporal dynamics and limit our understanding of its role in muscle atrophy progression. Longitudinal studies and real-time monitoring techniques can provide more comprehensive insights into the fluctuations of oxidative stress markers and their relationship with muscle wasting over time. In addition, considering the interplay between oxidative stress and other cellular processes, such as inflammation and mitochondrial function, is essential for a holistic understanding of their contribution to muscle atrophy [53]. Despite these challenges, integrating multiple approaches, using appropriate controls, and considering the context of the study can provide valuable insights into the role of oxidative stress in skeletal muscle atrophy. Advances in experimental techniques and the development more specific and sensitive markers will further enhance our understanding of oxidative stress in muscle health and disease.

## Conclusions

The current status of research on the role of oxidative stress in skeletal muscle atrophy reveals its significant impact and opens up avenues for future exploration. The review article has provided a comprehensive understanding of the mechanisms through which oxidative stress contributes to muscle wasting, shedding light on the activation of proteolytic systems, disruption of cellular homeostasis, and mitochondrial dysfunction as key factors. Furthermore, the systemic implications of oxidative stress on inflammation, hormonal imbalances, and overall health emphasize the need to consider its broader effects in the context of muscle atrophy. Moving forward, future research directions should focus on delving deeper into the complexities of oxidative stress in muscle atrophy. This involves investigating the specific ROS involved, elucidating novel signaling pathways, and uncovering the temporal and spatial aspects of oxidative stress in muscle tissue. In addition, exploring the intricate crosstalk between oxidative stress and other factors, such as inflammation, metabolic disturbances, and aging, will be essential to gaining a comprehensive understanding of the underlying mechanisms.
